# Changes in the HIV continuum of care following expanded access to HIV testing and treatment in Indonesia: A retrospective population-based cohort study

**DOI:** 10.1371/journal.pone.0239041

**Published:** 2020-09-11

**Authors:** Yane N. Tarigan, Richard J. Woodman, Emma R. Miller, Rudi Wisaksana, F. Stephen Wignall, Paul R. Ward

**Affiliations:** 1 College of Medicine and Public Health, Flinders University, Adelaide, Australia; 2 Department of Internal Medicine, Hasan Sadikin Hospital, University of Padjajaran, Bandung, Indonesia; 3 Bali Peduli Foundation, Denpasar, Bali, Indonesia; University of the Witwatersrand, SOUTH AFRICA

## Abstract

**Background:**

In 2013, the Indonesian government launched the strategic use of antiretroviral therapy (SUFA) initiative with an aim to move closer to achieving the UNAIDS 90-90-90 target. This study assessed the impact of SUFA on the cascade of HIV care.

**Methods:**

We performed a two-year retrospective population-based cohort study of all HIV positive individuals aged ≥ 18 years residing in two cities where SUFA was operational using data from HIV clinics. We analysed data for one-year pre- and one-year post-SUFA implementation. We assessed the rates of enrolment in care, assessment for eligibility for antiretroviral therapy (ART), treatment initiation, loss to follow-up (LTFU) and mortality. Multivariate Cox regression was used to determine the pre-to-post-SUFA hazard ratio.

**Results:**

A total of 2,292 HIV positive individuals (1,085 and 1,207 pre and post-SUFA respectively) were followed through their cascade of care. In the pre-SUFA period, 811 (74.6%) were enrolled in care, 702 (86.6%) were found eligible for ART, 485 (69.1%) initiated treatment, 102 (21%) were LTFU and 117 (10.8%) died. In the post-SUFA period, 930 (77%) were enrolled in care, 896 (96.3%) were found eligible for ART, 627 (70%) initiated treatment, 100 (16%) were LTFU and 148 (12.3%) dead. There was an 11% increase in the rate of HIV linkage to care (HR = 1.11; 95% CI 1.001, 1.22 p<0.05), a 13% increase in the rate of eligibility for ART (HR = 1.13, 95% CI 1.02,1.25, p<0.01) and a 27% reduction in LTFU (HR = 0.73, 95%CI 0.55, 0.97, p<0.05). Rates of ART initiation and mortality did not change.

**Conclusion:**

SUFA was effective in improving HIV care in relation to linkage to care, eligibility and ART retention. Therefore, the scale up across the whole of Indonesia of the SUFA currently in the form of a test and treat policy, with improvement in testing and treatment strategies is justified.

## Introduction

The expansion of HIV testing and treatment programs between 2010 and 2018 has contributed to a 9% decrease in the HIV infection rate and a 24% decline in AIDS-related deaths in the Asia Pacific region [1]. However, the extent to which efforts are being made across countries is still highly variable. Indonesia has seen a decline in HIV infections by 27%, but a 60% increase in AIDS-related deaths in the last decade [1]. A 2018 report reflected the lack of adequate testing and treatment within Indonesia, with only 51% of People Living with HIV (PLHIV) estimated to be aware of their HIV status, and only 33% receiving antiretroviral therapy (ART) [1].

The Treatment as Prevention (TasP) strategy for HIV control is a theoretically well grounded [2–4] and promising approach for accelerating the engagement to ART [5–7]. However, meaningful decreases in HIV incidence cannot occur unless a high uptake along the complete treatment cascade is achieved. Applying the evidence for structural, biomedical and behavioural interventions and contextualizing these into the local context could potentially improve the cascade of care [8, 9]. Whilst investigations have been performed in Sub-Saharan Africa [5–7, 10], relatively little is known about the combined effects of accelerated HIV testing and TasP along the full HIV continuum of care among general populations in countries with limited resources. One study examined the impact in Asia [11], however none have been conducted in the Southeast Asia region, an HIV-concentrated epidemic area where key affected population (KAP) are still important subgroup population in determining HIV dynamic in an area [12] and in which a multi-religious and multi-cultural context strongly influences behaviour.

The strategic use of antiretroviral therapy (SUFA) intervention was launched by the Indonesian government in 2013 [13]. The main strategies were *to identify* high-risk people, *to treat* eligible PLHIV and t*o retain* them in care. The program combined expanded access to HIV tests with a change in criteria for ART initiation to that of providing a fixed dose combination ARV treatment irrespective of CD4 count amongst specific risk populations [14, 15]. The aim of SUFA was to significantly reduce HIV morbidity, mortality and achieve progress towards the UNAIDS 90-90-90 goals. Eventually, this policy was expected to contribute to a reduction of HIV transmission in the long run. To date, there has been no longitudinal assessment of the impact of SUFA on the cascade of care.

We therefore used longitudinal data collected before and after the implementation of SUFA and assessed changes in the cascade of care outcomes including engagement to ART and death (death from any causes in an HIV infected patient) using data from HIV records from all HIV health care facilities in Medan and Batam, two of the SUFA sites. Engagement to ART in the Indonesian context, refers to the various stages of HIV care from enrolment, through eligibility, antiretroviral treatment initiation and retention. These detailed stages were the focus of our investigation.

## Material and methods

### Study design

**Fig 1:**
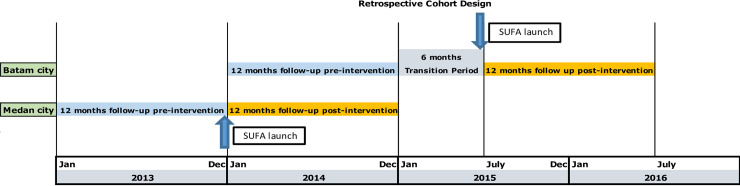
Retrospective study design of Pre and Post-SUFA intervention periods for the Medan and Batam cities. Two Pre-SUFA data and two Post-SUFA data periods were included. Each period and each city included follow-up for 12 months. The pre-SUFA intervention in Medan was for 26 Dec 2012–25 Dec 2013 and post-SUFA for 26 Dec 2013–25 Dec 2014. Pre-SUFA intervention in Batam was from 26 Dec 2013–25 Dec 2014 and post-SUFA 26 June 2015–25 June 2016.

We used a retrospective cohort study design with periods of one year pre-and one year post-implementation of SUFA intervention that included an overlapping follow-up in the two cities to assess the impact of the SUFA programme on HIV positive individuals who were enrolled in care; assessed for eligibility for ART; ART treatment initiation; ART retention; as well as death. Medan and Batam were purposefully chosen as the two comparison sites from the pilot SUFA sites and sites from the third phase of the SUFA implementation. Fig 1 describes the period of follow-up relative to the implementation of SUFA. SUFA was implemented in Medan on 26 December 2013, and in Batam on 26 Jun 2015. Both cities were followed for 12 months prior to and after their respective SUFA implementation dates, which included an overlapping follow-up period for the two cities of 12 months (pre-SUFA for Batam and post-SUFA for Medan). There was a six-month gap in Batam between pre- and post-SUFA as SUFA was launched in Batam six months later than the original plan. There were no new HIV policies implemented during that period. The pre SUFA follow-up period in Medan was 26 Dec 2012–25 Dec 2013 and in Batam was 26 Dec 2013–25 Dec 2014. The post SUFA follow-up period in Medan was 26 Dec 2013–25 Dec 2014 and in Batam was 26 Jun 2015–25 Jun 2016.

Both cities had similar characteristics regarding the HIV prevalence and both were considered to have concentrated epidemics [16], the prevalence of HIV among key affected populations in each area ((female sex workers (FSW), men who have sex with men (MSM), transgender people, people who inject drugs (PWID)); the availability of HIV treatment services including access to CD4 cell count testing [17]; socio-cultural and religious demographics [18]; HIV organisations; and the Human Development Index [19–23]. The study included all public and private hospitals and primary health centres in the two cities that had HIV cases detected within the period of interest (fourteen in Medan and six in Batam). All HIV patients were followed from the date of their HIV detection until the end of each 12-month follow-up period or their last observed date of follow-up if it occurred earlier, regardless of their stage of care in the cascade. Patients that received services in more than one clinic had their data combined.

### Ethical consideration

The study was approved by the Social and Behavioural Research Ethics Committee, Flinders University in Adelaide, Australia, (project approval number 7622) and The Health Research Ethics Committee, Faculty of Medicine, University of Padjajaran (KEPK- FK UNPAD) (project approval number 708/UN6. C.10/PN/2017). A letter of recommendation to conduct the study from the Directorate Prevention and Disease Control for Direct Transmission, Indonesian Ministry of Health was also obtained. All of the data obtained and analysed for the study were routine data extracted anonymously from medical records. The Indonesian MOH regulation 269 of year 2008 [24] provides the authority for the use of medical records for research purposes, when used for the benefit of the country, and also states that the use of de-identified medical data for research purposed does not require prior consent from patients. Therefore individual-level patient consent was not obtained.

### Study population

The study included all adults aged ≥ 18 years with an HIV record in a health facility and resided in Medan or Batam. A requirement for being included in the analysis of each care stage was entering the previous stage of care. The study was conducted between 21 July and 25 September 2017 in Medan and between 26 September and 30 October 2017 in Batam.

### The SUFA intervention

*To identify* HIV infection requires various interventions such as provider-initiated counselling and testing (PITC), voluntary counselling and testing (VCT), and mobile testing. VCT is defined as voluntary HIV testing and counselling initiated by an individual, while PITC is HIV testing and counselling initiated by health providers among patients in clinics or hospital wards. Mobile clinic testing refers to community outreach HIV testing, which was delivered outside clinics in communities where high-risk people live. *To treat* depends on two criteria: ART eligibility determined as either a CD4 count ≤350 cells/mm3 or immediate ART irrespective of CD4 count in specific high risk populations: FSW, MSM, transgender people, PWID, sero-discordant couples, pregnant women, and those with HIV/TB and HIV/HBV co-infections. *To retain in care* using a once daily tenofovir disoproxil fumarate (TDF) based fixed-dose combination (FDC) with lamivudine and efavirenz as first line treatments [14, 15].

### Pre-and post-SUFA policy differences

In the HIV test stage in the pre SUFA era, the target populations for PITC screening intervention were pregnant women, TB patients, key affected populations, sexually transmitted infection patients and their partners, and HIV-suspected patients. In the post SUFA era, the target populations were expanded to include co-morbid HIV- hepatitis patients, prisoners, high-risk men (e.g. intercity truck drivers, sailor men) and PLHIV partners. There were no strategy changes for VCT and mobile clinic interventions in either periods [14].

In the initiation stage of the pre SUFA era, the threshold for treatment eligibility was at the cut-off point of a CD4 count level ≤ 350 cells/mm3 [25]. While in the post SUFA era, the treatment indication was expanded to include membership in the specific populations with the addition of the criterion ‘irrespective of CD4 count’ [15, 26].

In retention stage of the pre SUFA era, the first line treatment used was zidovudine (ZDV) based, with treatment combining ZDV with two other loose drugs [25]. While in the post- SUFA era, utilisation of the TDF-based once daily fixed-dose combination became the first line drug regimen [15]. The single pill FDC is a strategy to improve retention of ART patients [14, 15, 27] by reducing pill burden and frequency.

Other innovations in post SUFA era included the decentralisation of ARV services to primary health centres, the integration of HIV testing services with other health services within clinics, and the simplification of the pre-ART procedure (such as role of laboratory examination from compulsory test to as necessary). The changes allowed task shifting for treatment, or counselling (delegation of tasks from highly specialised to less specialised health workers where necessary. Detailed of the pre-and post-SUFA intervention policies are shown in S1 Table.

### Data sources

Data were accessed from HIV clinics, private and public hospitals or primary health centres, that each used national standardized HIV report forms. All data were collected by clinic staff as part of routine HIV care [28]. Although medical records were used as the main source of data, other patient records were also scrutinised to reduce missing data. These data sources included the national HIV testing registry, counsellor books, summaries of HIV and ARV care, the register of pre-ART and ART, and other HIV registry records. The three-exact identity technique (name, home address and date of birth) was used when matching with medical records. If the data differed across medical records and the other registers, or appeared to be either invalid or unclear, the records officer was consulted for data verification.

### Outcomes

The five outcomes assessed were enrolment in care, readiness for ART, treatment initiation for ART, loss to follow up (LTFU) and death. S2 Table summarizes outcomes, definitions and estimations.

### Statistical analysis

Categorical variables were summarised using frequencies and percentages while continuous variables were summarised using median and range. Differences in the characteristics of participants between the pre- and post-SUFA periods were compared using chi-squared tests of association or Mann-Whitney U-tests as appropriate. Survival analysis was performed using Cox regression to assess the hazard ratio for the SUFA intervention on enrolment to care, eligibility for treatment, treatment initiation, LTFU and death. The follow-up period for each event was from time of becoming at risk for each event until the event occurrence or the end of the pre- or post-SUFA follow-up periods when individuals were censored. Summary statistics for the survival analysis were calculated including the number of individuals at risk for each event, the number of events, the total follow-up time, and the incidence rate for both the pre- and post-SUFA intervention periods. Cumulative incidences were visually described using the Kaplan Meir curve and the log-rank test was used to compare crude incidence rates for the pre- and post-SUFA cohorts for each event.

Multivariate Cox proportional hazard regression models were used to assess the independent association between the SUFA intervention and each event. Purposeful covariate selection was used in model building [29] with 4 separate adjustment models; model 1 was unadjusted, model 2 was adjusted for age and sex; model 3 was additionally adjusted for the district and time (month) and model 4 was additionally adjusted for potential known confounders including education attainment (no school/primary/high school/higher), employment status (working/not working) and transmission route (vaginal/anal/injected drug/drug transfusion/perinatal/other (bisexual/occupational exposure)). We also assessed possible interactions between SUFA and district. The proportional hazard assumption was assessed globally and for individual covariates. As a sensitivity analysis we also performed multiple imputation to account for any missing data (model 5) using chained equations with 30 imputed datasets [30]. All data analyses were conducted using Stata version 15.1 (StataCorp LLC, Texas).

## Results

**Fig 2:**
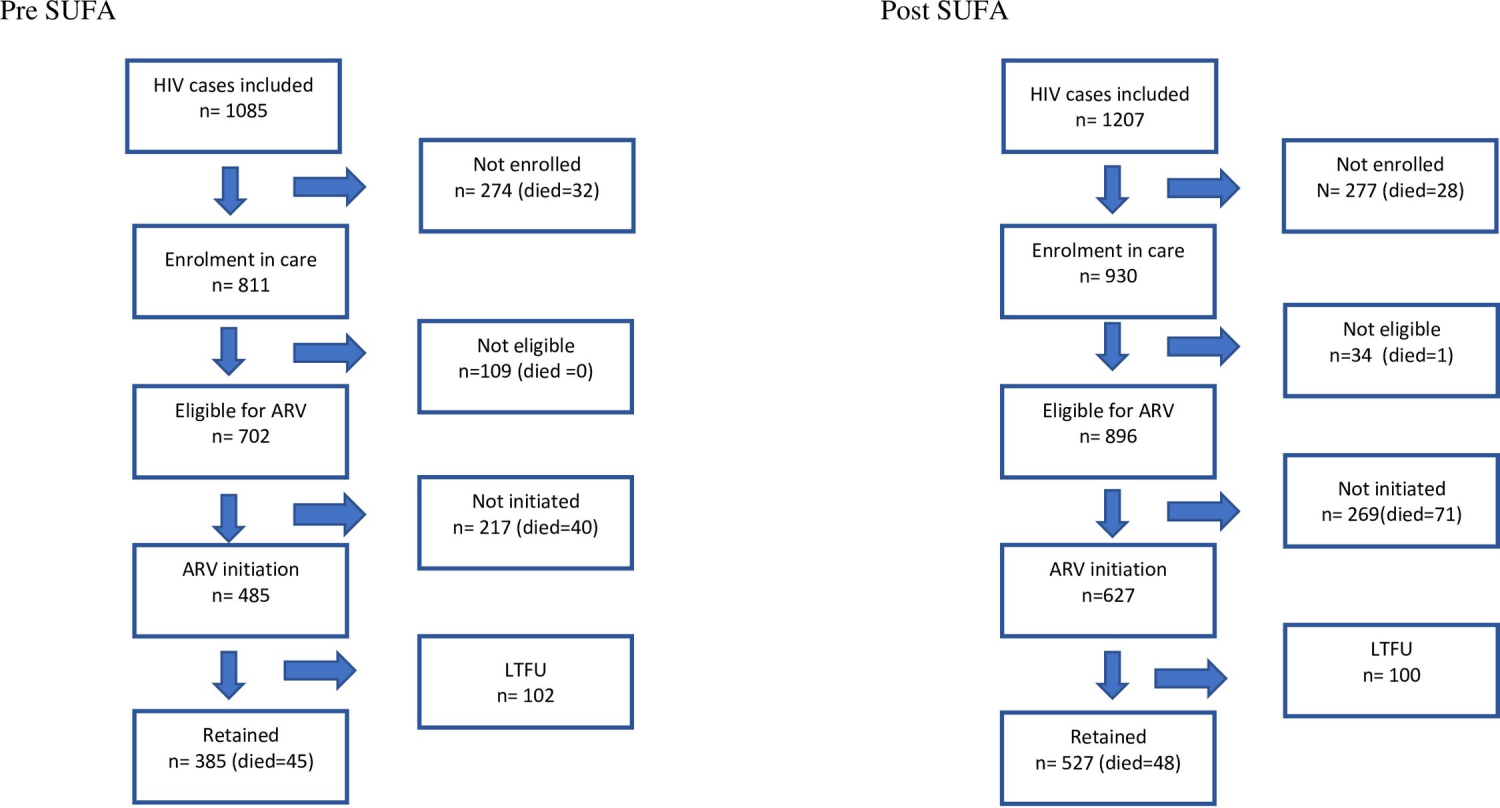
Flow diagram of data collection for cohort population, stratified pre-and post-SUFA.

A total of 2,292 individuals that were tested for and diagnosed with HIV were followed for linkage to HIV care, being eligible for ARV, receiving HIV treatment, LTFU during treatment and death. Of the 2,292 individuals, just over half (51.3%) were from Medan and just over half (52.7%) were in the post-SUFA period. Fig 2 describes the calendar time-flow of patients during the study. Approximately one quarter (24.1%) were followed in 2013, 50.5% in 2014 and 25.4% during 2015/2016.

Table 1 shows the demographic and clinical characteristics of the overall pre-and post-SUFA cohorts with additional detail in S3 Table. Of 1,085 HIV positive individuals in the pre-SUFA period, the median age was 32 years, 64.8% were male, 68.4% had completed at least high school education, 59.6% were in paid employment. PITC was reported as the main testing approach used (61.1%). Vaginal transmission was reported as the main mode of HIV transmission (68.6%). Of 1,207 individuals in the post- SUFA period, the median age was 32 years, 68.1% were male, 67.9% had completed at least high school education, 67.7% were in paid employment. The PITC was reported as the main testing approach used (60.2%). Vaginal transmission was reported as the main mode of HIV transmission (64.1%). Compared to the overall Indonesian population, the cohort population were older in age, and had a higher percentage of males and employed worker [31, 32].

**Table 1:** Demographic and clinical characteristics of the HIV population by pre- and post-SUFA periods.

Characteristics		Pre-SUFA n(%); median (IQR); N = 1085	Post-SUFA n(%); median (IQR); N = 1207	*P-Value
Age (**median) (IQR))**		32(28–38)	32(27–38)	0.760
Gender (**n (%))**	Male	703(64.8)	822(68.1)	0.078
	Female	363(33.5)	374(31)	
	Missing	19 (1.8)	11(0.9)	
Education attainment(**n (%))**	Lower(no school/primary school)	115 (10.6)	128(10.6)	0.952
	Higher (high school/higher)	742(68.4)	819(67.9)	
	Missing	228 (21)	260 (21.5)	
Employment (**n(%))**	Not worked	304(28)	267(22.12)	**<0.001 **
	Worked	647(59.6)	817(67.7))	
	Missing	134 (12.4)	123 (10.2)	
Risk transmission (**n(%))**	Vaginal	744(68.6)	774(64.1)	**<0.001 **
	Anal	139(12.8)	249(20.6)	
	Others (PWID/ bisexual/perinatal/blood transfusion/occupational)	89(8.2)	92(7.6)	
	Missing			
Test approach (**n(%))**	PITC	663(61.1)	725(60.2)	0.389
	VCT	285(26.3)	306(25.4)	
	Missing	137 (12.6)	176(14.6)	
Baseline Clinical staging (**n(%))**	I	92(11.3)	80(8.6)	**<0.05**
	II	106 (13.1)	126 (13.6)	
	III	360 (44.4)	457(49.1)	
	IV	128 (15.8)	165(17.7)	
	Missing	125 (15.4)	102 (11)	
Baseline CD4 cells/mm3 (**median) (IQR))**		120(32–265)	115(31–276)	0.885
Baseline CD4 cells/mm3 (**n(%))**	< 350	493 (60.8)	601(64.6)	0.234
	>350	88(10.9)	96 (10.3)	
	Missing	230 (28.4)	233 (25.1)	
Treatment Indication (**n(%))**	Baseline Clinical staging III or IV or CD4 cells count ≤ 350/mm3	468(96.5)	559(89.2)	**<0.001**
	SUFA criteria and others	16(3.3)	60(9.6)	
	Missing	1(<1)	2(<2)	
ARV drug initiation (**n(%))**	ZDV (300) + 3TC (150) + NVP (200	259(53.4)	113(18)	**<0.001**
	ZDV (300) + 3TC (150) + EFV (600)	85(17.5)	32(5.1)	
	TDF (300) + 3TC (150) + NVP (200)	34(7)	34(5.4)	
	TDF (300) +3TC (150) +EFV (600)	90(18.6)	101(16.1)	
	TDF (300) +3TC (300) +EFV (600)	7(<2)	334(53.3)	
	Others	3(<1)	3(<1)	
	Missing	7(<2)	10(<2)	
District (**n(%))**	Medan	552 (50.9)	624(51.7)	0.694
	Batam	533 (49.1)	583(48.3)	
Time (**n(%))**	2013	552(50.9)	0	**<0.001 **
	2014	533 (49.1)	624(51.7)	
	2015/16	0	583(48.3)	
Enrolment (**n(%))**	Enrolled in	811 (74.6)	930 (77)	0.329
	Not enrolled	274 (25.3)	277 (23)	
Eligibility (**n(%))**	Eligible	702 (86.6)	896(96.3)	**<0.001**
	Not eligible	109 (13.4)	34(3.7)	
Initiation (**n(%))**	Initiation	485 (69.1)	627(70)	0.719
	Not initiation	217 (30.9)	269 (30)	
LTFU (**n(%))**	LTFU	102 (21)	100(16)	**<0.05**
	Not LTFU	383 (79)	527(84.1)	
Death (**n(%))**	Dead	117 (10.8)	148 (12.3)	0.335
	Not dead	968(89.2)	1.059(87.7)	

^**1**^Pearson chi-square test, Mann-Whitney test or t-test as appropriate.

Among the 1,085 HIV positive individuals in the pre-SUFA period, 811 (74.6%) were enrolled in care, 702 (86.6%) were found eligible for ART, 485 (69.1%) received treatment for the first time, 102 (21%) were LTFU and 117 (10.8%) died including 45 who died after initiating treatment (S4 Table). Among the 1,207 HIV positive individuals in the post-SUFA period, 930 (77%) were enrolled in care, 896 (96.3%) were found eligible for ART, 627(70%) were initiated to treatment, 100 (16%) were LTFU and 148 (12.3%) died, including 48 who died after commencing ARV (S4 Table).

**Fig 3:**
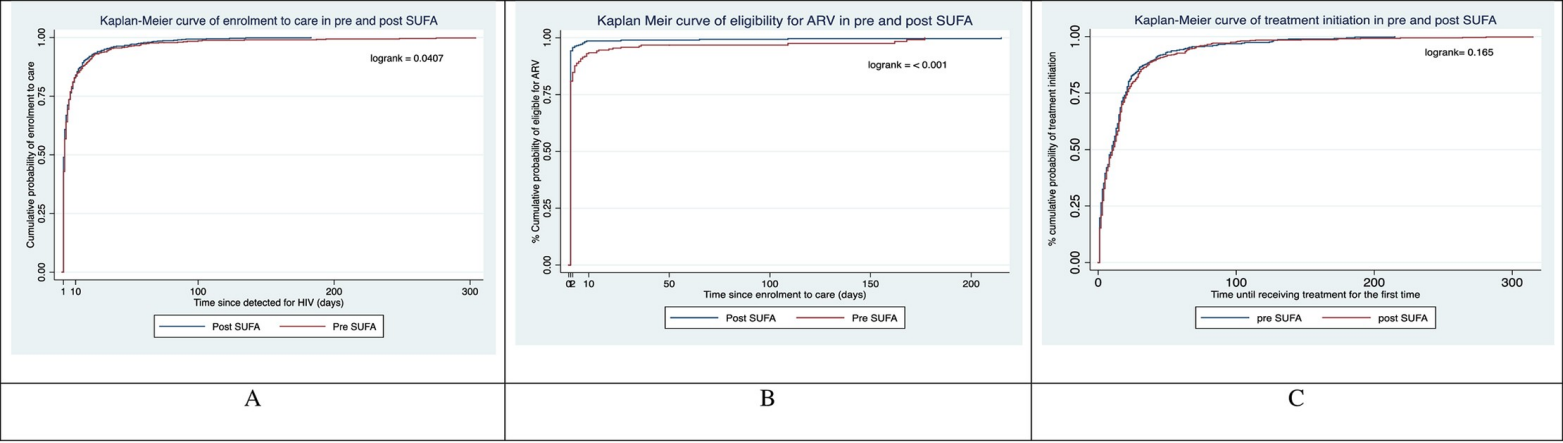
Kaplan Meir plots and log-rank statistics for A) enrolment in care among people who were detected, B) eligibility for ARV among people who enrolled in care, and C) treatment initiation among people observed eligible for ARV, stratified by pre-and post-SUFA intervention periods.

Although the median time from HIV detection to enrolment in care was one day (IQR 0–5 days) in both periods, the rates of enrolment differed between pre- and post-SUFA periods (p = 0.0407) (Fig 3). In the fully adjusted Cox regression analysis, enrolment in the post-SUFA intervention period was also significantly higher than in the pre-SUFA intervention period (HR = 1.10; 95% CI 1.001, 1.22 p<0.05) and remained so after using multiple imputation for missing data (HR = 1.11; 95% CI 1.004, 1.22 p<0.05) (Table 2).

**Table 2:** Univariate and multivariate Cox model for enrolment in care, eligibility for ART, treatment initiation, LTFU, and death during pre -SUFA and post-SUFA intervention periods.

Outcome	Period	Subjects (N)	Events (N)	Person-days of follow-up	Event rate (events/1000 person-days)	HR model 1 (95%CI)	HR model 2 (95%CI)	HR model 3 (95%CI)	HR model 4 (95%CI)	HR model 5 95% CI)
Enrolment to care	Pre-SUFA	1,085	811	5892	137.6	Ref	Ref	Ref	Ref	Ref
	Post-SUFA	1,207	930	5232	177.8	1.08 (0.99, 1.19)	1.08 (0.98 1.18)	1.08 (0.98, 1.19)	1.10* (1.001,1.22)	1.11* (1.004,1.22)
Eligibility for ART	Pre-SUFA	811	702	1872	375.0	Ref		Ref	Ref	Ref
	Post-SUFA	930	896	1403	638.6	1.16** (1.05, 1.28)	1.16** (1.05, 1.28)	1.16** (1.05, 1.28)	1.11 (0.997, 1.24)	1.13** (1.02,1.25)
Treatment initiation	Pre-SUFA	485	702	7820	55.4	Ref	Ref	Ref	Ref	Ref
	Post-SUFA	627	896	11326	62.0	0.92 (0.82, 1.04)	0.92 (0.82, 1.03)	0.92 (0.81, 1.03)	0.90 (0.80, 1.02)	0.90 (0.80, 1.01)
LTFU	Pre SUFA	102	485	49862	2.0	Ref	Ref	Ref	Ref	Ref
	Post SUFA	100	627	69258	1.44	0.72* (0.54, 0.94)	0.71* (0.54, 0.93)	0.71* (0.54, 0.94)	0.78 (0.57, 1.05)	0.73* (0.55, 0.97)
Death	Pre-SUFA	117	1,085	77776	1.90	Ref	Ref	Ref	Ref	Ref
	Post SUFA	148	1,207	55779	2.10	1.00 (0.78, 1.27)	1.00 (0.79, 1.28)	1.00 (0.78, 1.27)	0.94 (0.72, 1.21)	1.03 (0.80, 1.31)

* = Statistically significant at p value <0.05

**Statistically Significant at p value <0.01; Enrolment to care: Model1 = unadjusted; Model 2 = adjusted for age and sex; Model 3 = adjusted for age, sex, district, time; Model 4 = model adjusted for age, sex, district, time, employment status; Model 5 = model adjusted to covariates in model 4 and using 30 imputed datasets. Eligibility for ARV: model 4 = adjusted for age, sex, district, time, education attainment. Treatment initiation: model 4 = model adjusted for age, sex, district, time, employment status. LTFU: model 4 = adjusted for age, sex, district, time, education attainment. Death: model4 = adjusted for age, sex, district, time, type of transmission.

The majority of individuals in both the pre-and post-SUFA periods were eligible for ART based on either a CD4 count ≤ 350 or being at a WHO clinical staging of 3 or 4 (S5 Table). The median time from enrolment in care to being assessed as eligible for ART was less than one day (IQR 0–0) in both periods, but was still significantly shorter in the post-SUFA period in log-rank analysis (p<0.001) (Fig 3). In univariate Cox regression, the SUFA intervention was strongly associated with faster eligibility for ART (HR = 1.16; 95% CI 1.05, 1.28, p <0.01) and became non-significant in the fully adjusted model (HR = 1.11; 95%CI 0.997, 1.24, p = 0.056). In the same model with multiple imputation, the association became significant (HR = 1.13, 95% CI 1.02,1.25, p<0.01) (Table 2).

S6 Table shows the proportion of subjects with treatment initiated based on their clinical condition/CD4 count or otherwise. In the pre-SUFA period, 96.5% of patients received treatment on the basis of their advanced clinical condition and 89.2% in the post-SUFA period. The median time until receiving ART for the first time was not different between the pre- and post-SUFA periods 9 days (1–19 days) vs. 10 days (2–20 days) respectively, log-rank p = 0.165) (Fig 3). Similarly, time until receiving first ART was not associated with SUFA in either univariate or multivariate analysis (Table 2).

In regard to LTFU after initiation of treatment, the majority of individuals in the pre-SUFA period (53.4%) received a free triple combination of ZDV (300) + 3TC (150) + NVP (200) twice daily, while the majority of the post-SUFA cohort (53.3%) received a fixed drug combination of TDF (300) +3TC (300)+EFV (600) or named SUFA drug in the post-SUFA group (p<0.001) once daily (Table 1). In univariate analysis, the SUFA intervention was associated with a 28% reduction in LTFU after commencing treatment (HR = 0.72, 95% CI 0.55, 0.95, p<0.05). In the fully adjusted model the association was not significant in the analysis with missing data and significant in the fully adjusted model with multiple imputation (HR = 0.73, 95%CI 0.55, 0.97, p<0.05) (Table 2).

There were 117 individuals that died (11%) in the pre-SUFA cohort and 148 that died in the post-SUFA cohort (12.3%) (Table 2). The percentage of individuals that died after initiation of treatment was lower in post-SUFA compared to pre-SUFA (34.7% versus 48.9%, p = 0.012). However, the SUFA intervention was not associated with overall mortality in either univariate analysis, multivariate analysis or after multivariate analysis with multiple imputation (Table 2).

## Discussion

The SUFA intervention combined a revision of treatment criteria for persons with HIV to TasP and a change from a twice-daily to a once daily fixed drug combination. In addition to these patient level changes, the SUFA intervention also implemented changes at the community, practice and policy level including HIV stakeholder networking, health providers and community training, community- and facility-based HIV testing and counselling. Overall, this study shows that SUFA was associated with an 11% higher rate of linkage to care, a 13% increase in those being eligible for ART and a reduction in the loss-to-follow up of those after treatment initiation of 27%. However, the policy intervention did not demonstrate any meaningful improvements in time to treatment initiation or mortality. Together these changes demonstrate a meaningful step forward in Indonesia’s aim to achieve the UNAIDS 90-90-90 targets.

### Enrolment to care

The moderate 11% increased rate of enrolment to care amongst HIV positive persons was likely the result of an improvement in services efficiency where individuals could be immediately enrolled in care after testing positive within the same health facility. SUFA thereby addresses some of the known structural and physical barriers including the need for simultaneous clinical services [33] and social aspects including transportation costs. PITC was the dominant testing approach used both prior to and after the SUFA intervention, compared to VCT. PITC is conducted in health care clinics, thereby facilitating patient access to HIV care, treatment and support services [34, 35] as well as reducing the potential for HIV stigma and the time for test completion [36]. Wu, Zhao [11] observed the median time of linkage to care was one day and zero days after the simplified procedure. McNairy, Lamb [9], observed a mean time for linkage to care of 2.5 days post-intervention. In Africa however, where they utilised mostly community based HIV counselling and testing, the long period between being diagnosed for HIV and becoming enrolled to care is still of huge concern, with reported rates of only 47.5% linked to care within six months of referral in South Africa [5] and 34% after six months in Swaziland [37]. As highlighted by Ayieko, Petersen [38], it may be more difficult to link PLHIV identified outside clinics into care than when PLHIV are found in facilities that already provide comprehensive HIV care services.

### Eligibility for ARV

The use of the new SUFA eligibility criteria for treatment, which includes all HIV specific persons irrespective of CD4 count, was associated with a 13% increase in the rate of eligibility for ART. The finding is comparable to studies investigating a multicomponent intervention (POC, accelerated eligibility assessment and treatment, applied mobile phone reminders for appointments, provided health educational packages, and non-cash financial incentives) which reported increases in eligibility for ARV of 18% [9] and 24% [8] following intervention. This is despite both studies using a CD4 count ≤350 cells/mm3 as the eligibility criterion for ARV. The impact of SUFA was not as large as the doubling anticipated with the expanded criteria [39]. The reason for this may be because there was a substantial number of asymptomatic HIV-infected individuals who might have benefitted from the new criteria but were not identified. The majority of individuals in both the pre- and post-SUFA cohorts were diagnosed at an advanced stage of HIV infection, with a median baseline CD4 count of 115 cells/mm3, 64.6% with CD4 counts ≤350, and 66.8% at clinical stage 3 or 4 post-SUFA. Hospital-based testing strategies for HIV generally find more delayed diagnoses [35, 40, 41] compared to community-based testing strategies [35, 41]. SUFA’s main testing strategy targets hospital and clinical settings (PITC), leading to more patients in advanced stages of HIV progression. The contribution of SUFA to finding early cases was small (median CD4 count was ≤ 200 in the post-SUFA period), indicating a more comprehensive testing strategy is still required in order to capture those outside of healthcare facilities.

### Treatment initiation

Although the number of people initiating ART was projected to grow substantially based on studies elsewhere [39] SUFA did not improve ART initiation despite the higher numbers of those eligible. Studies in Swaziland [9], Kwazulu Natal and Uganda [42], South Africa [43] and Zambia [44] found similarly disappointing results. After applying multiple interventions that targeted several stages in the cascade of care, treatment uptake did not improve, remaining at a relatively low 37% [42] and 22% [43].

A potential factor related to limited treatment uptake was that the multiple steps and long procedure prior to treatment initiation were not significantly improved. Januraga et al. [45] highlighted the existence of similar challenges. Complex blood testing algorithms and the requirement for multiple counselling sessions were two policies that were simplified after SUFA in the new ARV guidelines [15]. A simplified blood test algorithm based on clinical condition and CD4 count not being a requirement for initiation may not have been followed. The second supposed simplification was the stipulation of four compulsory adherence to treatment counselling sessions, rather than the previous ad hoc frequency of sessions [14]. Prior to initiation to ARV, patients must undertake adequate and appropriate treatment counselling, after which their possible adherence level is assessed and predicted based on information collected from the counselling session. However, this procedure potentially leads to subjectivity in interpretation of the patient’s knowledge and adherence level, thus potentially delaying the treatment.

A prospective cohort study in four different areas in Indonesia found comparable results to ours with a median time from study registration to initiation of ART treatment of seven days [45] compared to the 9 to 10 days in this study. Together, this indicates that the Indonesian health system has reduced the time from diagnosis to receiving treatment, at a time when other areas of the world still experience huge challenges. The median time from diagnosis to ART treatment have been reported elsewhere as 34 days [37], 118 days [46], 6 months [6], 22 weeks [42], and 265 days [5]. However, these populations may have faced more significant barriers in linking PLHIV detected using community-based HIV counselling and testing settings compared to this study in which the majority of PLHIV were detected in health care facilities.

Nevertheless, 2014 SUFA progress report by Ministry of health (unpublished report) showed that HIV testing in 13 SUFA pilot demonstration sites increased after its implementation. If a higher number of persons with HIV were therefore potentially captured due to increased testing, we could assume that the overall rate of ART uptake in the two cities increased. However, for confirmation, this would require access to data on testing rates which was not captured in this study.

### LTFU

Introduced to facilitate compliance with treatment, an important component of the SUFA intervention, was the single dose fixed triple combination of HAART, which is regarded as safe and well-tolerated [25, 47]. Most individuals in the post SUFA period used the once daily treatment, which was likely to be the cause for the significant improvement in retention (i.e. reduction in LTFU). Amongst persons LTFU, the majority in the post-SUFA period used the SUFA drug regimen, whilst the majority of those LTFU in the pre-SUFA period used the dose-free combination of ZDV (300) + 3TC (150) + NVP (200) twice per day. In agreement with the previous findings of Amico [48], our study indicates that simpler drug regimens might be a reason for the reduction in LFTU incidence after initiation to treatment. Other important factors contributing to treatment retention are readiness to start long life treatment and adherence to serial counselling. Prior to SUFA, counselling adherence was required in Indonesia before treatments were provided to HIV eligible patients. In the post-SUFA era, patients were additionally required to receive routine treatment reminders and to provide a written statement of readiness to take life-long treatment [14, 26]. Koenig observed a 99% readiness amongst patients to begin life-long treatment [49]. Whilst we did not specifically measure readiness, it is likely patients would have similar readiness given their need to also overcome barriers such as perceived stigmatisation from providers, and a lack of laboratory and transportation costs in the process of being linked to care and receiving treatment.

### Mortality

The structural interventions that expanded access to HIV testing and ART treatment in SUFA did not impact on mortality in the year following its implementation. Global evidence has suggested that treating HIV infected people before their CD4 counts are lowered could decrease clinical disease progression and mortality by up to 44% [2–4, 50]. Although the aim of SUFA was to capture HIV persons at a healthier stage and provide immediate treatment, with the expectation of reducing morbidity and mortality [39] delayed diagnosis (defined as CD4 counts <350) occurred in similar proportions pre- and post-SUFA which could partly explain the lack of any apparent effects of SUFA on mortality. In this study, ART contribution to mortality is likely minimal given late entry into treatment with many patients already severely ill at treatment initiation and dying early in their care. Early diagnosis remains a crucial challenge in order to reduce mortality as seen previously in Indonesia [45] and elsewhere [11, 51–53]. Possible reasons for the lack of change in mortality, despite reduced LTFU, include in addition to delayed diagnosis, that the study is restricted to a one-year cohort follow up, and a longer follow- up is needed to examine the LTFU effect on mortality.

### Challenges towards the first 90 (90% of all PLHIV aware of their HIV status)

Our data suggest that the applied testing strategy that identified 2,292 and enrolled 1,741 of PLHIV of whom 1,097 approximately two-thirds (63%) were in the late stage of HIV disease. However, the estimated proportion of PLHIV that are aware of their HIV status in Indonesia has recently been reported as 42% (UNAIDS 2017). As such, many PLHIV, particularly in the asymptomatic stage were still not captured by the SUFA testing strategy, and might remain unsupported outside of the health care facilities. Thus, fundamental revisions of the testing strategy from its current design to a more effective combination of facility-based, community-based testing and self-testing, addressing all barriers to diagnosis, including fear of stigmatisation (a common reason in Indonesia for refusal to be tested) [54], remains a crucial element to be addressed for success of the current test and treat strategy [55]. Nonetheless, implementation science research is suggested for Indonesia to design the most appropriate combination of facility-based, community-based and self-HIV testing while rapidly bringing programs to scale.

### Challenges towards the second 90 (90% of all people diagnosed HIV receiving treatment)

Of the 96.3% of HIV persons eligible for ARV treatment post SUFA, 9.5% were added through the new SUFA criteria. Put differently, most identified cases would have already been eligible under the old policy. Further, approximately 30% of those eligible did not receive treatment. While the reasons for this were not explored in the project, it is possible that economic issues such as cost of laboratory tests, administrative and transportation costs, lengthy, and burdensome and multiple clinic visits prior to treatment initiation stages contributed to this failure [11, 49, 54, 56] as well as HIV stigmatization by providers [26].

The issue of multiple and burdensome visits are among the main reasons for drop out prior to receiving treatment [56], although this is lower in Indonesia than other contexts, where up to six visits are required before ART initiation [11, 56]. In Indonesia, the number of visits a patient is required to make depends on the patient’s condition with respect to opportunistic infections (OI) [15], prediction of adherence [14], and whether the patient has to be referred to other health care facilities to obtain comprehensive HIV care and treatment. These conditions sometimes lead to subjective interpretation of the appropriate time to start the treatment. A same day test and treat approach is effective in tackling this problem [49] and whilst this is now allowed in Indonesia, many procedures and barriers in the pre-ART practice still need simplification or elimination. Nevertheless, research must be conducted to assess procedures that hamper the treatment process, and based on the evidence, standardized procedures developed to avoid subjective interpretation and monitored in order to maintain a high quality of patient care and treatment.

### Challenges towards the third 90 (90% of all people receiving ARV becoming virally suppressed)

Although the SUFA intervention could show effectiveness in reducing LTFU by 27% after treatment initiation, non-initiation of treatment still limited the success in achieving the third 90. The third target has been found to be a similarly strong challenge elsewhere. In the hyperendemic setting of South Africa, an intervention that was effective in achieving earlier stage treatment, only 64% of subjects were retained and became virally suppressed at ten months [56]. In the low generalized epidemic setting in Haiti, of 79.8% of PLHIV retained to ARV, only 53% were retained and became virally suppressed at 12 months [49].

### Strengths and limitations

To our knowledge, this is one of the first studies to assess the effects of a multicomponent HIV testing and treatment intervention at multiple stages on the cascade of care and the first such study in Southeast Asia. The study investigated a large population of HIV positive individuals and used matched cities to help account for differences in the time of the implementation of the intervention. Data collection was based on standardised medical record review rather than subjective self-report [57]. Since all HIV cases reported in the two areas were included in the study, our findings can be generalised to similar HIV positive populations in Indonesia. Similarly, data were obtained in the real-world setting rather than in controlled laboratory settings. The findings are, therefore, likely to be more generalisable than those obtained from carefully controlled studies.

Our findings should also be interpreted carefully due to several limitations. The use of non-probability sampling to select the two locations might limit the generalisability findings to other districts in Indonesia given the variety of the region’s social, cultural, economic, and epidemiological characteristics as well as differences in health system capacity. In addition to the confounders that were included in our model, there may have been other unobserved confounders related to the locations, the time periods that were used for study as well as subjects’ clinical profiles that could not be adjusted for. Also, a longer period of observation prior to the start of SUFA would have helped reduce the possibility of a regression to the mean effect caused by introducing SUFA immediately after a period with unusually poor enrolment and retention. Unfortunately, we did not have individual patient level data for longer than the periods provided in this study. The study also used routinely collected clinical data that may sometimes have been incomplete or inaccurate. However, incorrect data can be assumed minimal since staff had received extensive training in entering data and used standardised forms for recording. Where data were incomplete, the researcher consulted the officer in charge or searched other records to complete the patient’s care history. In addition, the increase in significance e.g. in eligibility for ART were primarily the result of an increase in statistical power from using a full dataset after imputation rather than any change in the estimated effects. The conclusions obtained from multiply imputed analyses were generally similar to those obtained from those in the complete-case analysis. The point estimates for the hazard ratio’s were only changed slightly in each case suggesting that the missing data were missing completely at random or missing at random and was not due to any bias caused by data being missing not at random. Finally, we did not analyse viral load suppression data in the study due to insufficient data. We used LTFU or retention in care as a proxy indicator for the third 90 (virally suppressed), so an assumption of SUFA performance and challenges towards the third 90 could be explained.

## Conclusions

In conclusion, our study has revealed the strengths, weaknesses and challenges still faced by the Indonesian health system to improve the HIV continuum of care. The main effects of the SUFA intervention were quicker enrolment to care, more rapid identification of those eligible for treatment and better retention to treatment. However, there was no evidence that applying the policy of providing treatment irrespective of CD4 count contributed to more persons being eligible for treatment and reducing mortality. Developing and implementing strategies that recruit the less reachable populations and also address the multiple barriers in the various stages of the HCC is crucial. While the same day test and treat is a worthwhile intervention, for optimal effect, standardised guidelines for moving from testing to same day treatment initiation must be in place for the test and treat policy to become adopted as common practice. Whilst SUFA can be described as an overall step forward, sustained efforts are required if the UNAIDS 90-90-90 goal and a significant reduction of HIV transmission in 2030 can be seen as realistic for Indonesia.

## Supporting information

S1 Table Comparison of HIV intervention strategy in health facilities at Pre- and post-SUFA. (DOCX)

S2 Table Events, definitions and dates used in estimating follow-up time. (DOCX)

S3 Table Demographic and clinical characteristics of overall cohort population and sub-population of Enrolment in care, Eligibility for ARV, Treatment initiation, LTFU, Death stratified by Pre- and Post-SUFA intervention group. (DOCX)

S4 Table Deaths before and after receiving treatment amongst newly diagnosed HIV cases pre and post SUFA intervention. (DOCX)

S5 Table Eligibility criteria used amongst eligible persons. (DOCX)

S6 Table Treatment indication amongst patients initiated for treatment. (DOCX)
