# Supplementary material for: Changes in the HIV continuum of care following expanded access to HIV testing and treatment in Indonesia: A retrospective population-based cohort study
Source: PLoS One. 2020 Sep 11;15(9):e0239041. doi: 10.1371/journal.pone.0239041 (PMC7485792; doi:10.1371/journal.pone.0239041)
Supplement: S1 Table — (DOCX) [file pone.0239041.s001.docx]

Supplementary table 1. Comparison of HIV intervention strategy in health facilities at Pre- and post-SUFA

| **Cascade stage** | **Intervention** | **Pre-SUFA*** | **Post-SUFA**** | **Post-SUFA new elements** |
| --- | --- | --- | --- | --- |
| HIV Test | Screening strategy | VCT | VCT | - |
|  |  | PITC  Population target:  Pregnant women, TB patients, key affected population, STI patients and their partners, HIV suspected patients | PITC  Population target:  Pregnant women, TB patients, key affected population (every 6 months), STI patients, Hepatitis patients, prisoners, high-risk men, PLHIV partners, HIV suspected patients | Additional Target population |
|  |  | Mobile clinic | Mobile clinic | - |
|  | Testing procedure | Rapid test using 3 reagents or ELISA | Rapid test using 3 reagents or EIA or Western blot | - |
|  | Clinic system | Non-integrated | Integrated within other services in clinics | Integration system |
|  |  | Not specified | Compulsory providing HIV screening in all health clinics | Larger scale and expansion of testing services |
| Enrolment and determination of eligibility for ARV | WHO clinical staging assessment | Applied | Applied | - |
|  | Laboratory examination | CD4 count level (to initiate ART and control health progress), serum creatinine, SGPT/SGOT | CD4 count level (to control for health progress), serum creatinine or eGFR, SGPT, HB (if available) | Role of laboratory examination for treatment initiation from compulsory test before treatment to as necessary |
|  | Cotrimoxazole | Purpose given as a proxy adherence for those who have CD4 count < 200 cell/mm3 or WHO staging 2,3,4 | Given as indicated | - |
|  | Treatment counselling | Frequency of treatment as indicated; Compulsory provided by certified counsellors | Frequency of treatment maximum 4 times; Provided by certified counsellors or by trained health providers | Task shifting allowed |
| Initiation | Treatment indication for: |  |  |  |
|  | PLHIV | CD4 count ^2^ ≤ 350 cells/mm3 or WHO clinical stadium^3^ 3 or 4 whatever level of CD4 count | CD4 count ^2^ ≤ 350 cells/mm3 or WHO clinical stadium^3^ 3 or 4 whatever level of CD4 count | - |
|  | PLHIV from KAP | CD4 count ^2^ ≤ 350 cells/mm3 or WHO clinical stadium^3^ 3 or 4 whatever level of CD4 count | Irrespective CD4 count level and WHO clinical staging | Criteria changes to universal treatment |
|  | PLHIV from generalized epidemic | CD4 count ^2^ ≤ 350 cells or WHO clinical stadium^3^ 3 or 4 whatever level of CD4 count | Irrespective CD4 count level and WHO clinical staging | Criteria changes to universal treatment |
|  | Partner HIV positive of sero-discordant couple | CD4 count ^2^ ≤ 350 cells/mm3 or WHO clinical stadium^3^ 3 or 4 whatever level of CD4 count | Irrespective CD4 count level and WHO clinical staging | Criteria changes to universal treatment |
|  | Pregnant women | Irrespective CD4 count level and WHO clinical staging | Irrespective CD4 count level and WHO clinical staging | - |
|  | TB/HIV | Irrespective CD4 count level | Irrespective CD4 count level and WHO clinical staging | - |
|  | Hepatitis B | Irrespective CD4 count level | Irrespective CD4 count level and WHO clinical staging | - |
| Retention | First line ARV treatment | AZT or TDF based according to patients health conditions | TDF (fixed drug combination) | Changes of first line treatment regimen to TDF based Fixed drug combination once daily |
|  |  | To treat to retain PLHIV in ARV were only in hospitals only | Decentralized the services to primary health centers | Decentralization |
