# Supplementary material for: Changes in the HIV continuum of care following expanded access to HIV testing and treatment in Indonesia: A retrospective population-based cohort study
Source: PLoS One. 2020 Sep 11;15(9):e0239041. doi: 10.1371/journal.pone.0239041 (PMC7485792; doi:10.1371/journal.pone.0239041)
Supplement: S2 Table — (DOCX) [file pone.0239041.s002.docx]

Supplementary table 2. Events, definitions and dates used in estimating follow-up time

| **Event** | **Definition** | **Date of follow-up** |
| --- | --- | --- |
| Enrolment in care | Patient linked to care support and treatment unit | Date first documented for WHO clinical staging assessment or CD4 count exam or received cotrimoxazole prophylaxis |
| Eligibility for ART | Patient entitled for ART | Date first identified as eligible for ART treatment |
| Initiation of ART | Patient initiated for ART | Date of starting ART |
| Loss to follow-up post ART | Patient non-attendance for at least 3 months since last drug refill | Date at 3 months since last visit for drug refill |
| Death | Observed death date | Date of observed death |
