# Supplementary material for: Changes in the HIV continuum of care following expanded access to HIV testing and treatment in Indonesia: A retrospective population-based cohort study
Source: PLoS One. 2020 Sep 11;15(9):e0239041. doi: 10.1371/journal.pone.0239041 (PMC7485792; doi:10.1371/journal.pone.0239041)
Supplement: S3 Table — (DOCX) [file pone.0239041.s003.docx]

Supplementary table 3. Demographic and clinical characteristics of overall cohort population and sub-population of Enrolment in care, Eligibility for ARV, Treatment initiation, LTFU, Death stratified by Pre- and Post-SUFA intervention group

| **Characteristics** | | **Sub-pop 1 (Enroll)**  **A** | | **Sub-pop 2 (Eligible)** | | **Sub-pop 3 (Initiation)** | | **Sub-pop 4 (LTFU)** | | **Sub-pop 5 (Death)** | |  |  |  |  |  |
| --- | --- | --- | --- | --- | --- | --- | --- | --- | --- | --- | --- | --- | --- | --- | --- | --- |
|  |  | Pre-SUFA n(%); median  (IQR) N=1085 | Post-SUFA n(%); median (IQR) N=1207 | Pre-SUFA n(%); median  N=811 | Post-SUFA n(%); median (IQR N=930 | Pre-SUFA n(%); median (IQR) N=702 | Post-SUFA n(%);median (IQR N=896 | Pre-SUFA n(%);median (IQR)  N=485 | Post-SUFA n(%);median (IQR N=627 | Pre-SUFA n(%); median (IQR)  N=1085 | Post-SUFA n(%); median (IQR N=1207 | P1* | P2* | P3* | P4* | P5* |
| Enrolment | Enrolled in | 811 (74.6) | 930 (77) | --- | --- |  |  |  |  |  |  |  |  |  |  |  |
|  | Not enrolled | 274 (25.3) | 277 (23) | --- | --- |  |  |  |  |  |  |  |  |  |  |  |
| Eligibility | Eligible |  |  | 702 (86.6) | 896(96.3) |  |  |  |  |  |  |  |  |  |  |  |
|  | Not eligible |  |  | 109 (13.4) | 34(3.7) |  |  |  |  |  |  |  |  |  |  |  |
| Initiation | Initiation |  |  |  |  | 485 (69.1) | 627(70) |  |  |  |  |  |  |  |  |  |
|  | Not initiation |  |  |  |  | 217 (30.9) | 269 (30) |  |  |  |  |  |  |  |  |  |
| LTFU | LTFU |  |  |  |  |  |  | 102 (21) | 100(16) |  |  |  |  |  |  |  |
|  | Not LTFU |  |  |  |  |  |  | 383 (79) | 527(84.1) |  |  |  |  |  |  |  |
| Death | Dead |  |  |  |  |  |  |  |  | 117 (10.8) | 148 (12.3) |  |  |  |  |  |
|  | Not dead |  |  |  |  |  |  |  |  | 968 (89.2) | 1.059 (87.7) |  |  |  |  |  |
| Age |  | 32(28-38) | 32(27-38) | 32 (28-38) | 33(27-39) | 32 (28-38) | 33(27-39) | 32 (28-38) | 33(28-39) | 32(28-38) | 32(27-38) | 0.760 | 0.351 | 0.702 | 0.872 | 0.760 |
| Gender | Male | 703(64.8) | 822(68.1) | 545(67.2) | 657(70.7) | 471(67.1) | 640(71.4) | 322(66.4) | 448(71.6) | 703(64.8) | 822(68.1) | 0.078 | 0.184 | 0.110 | 0.122 | 0.078 |
|  | Female | 363(33.5) | 374(31) | 266(32.8) | 272(29.3) | 231(32.9) | 255(28.5) | 163(33.6) | 178(28.4) | 363(33.5) | 374(31) |  |  |  |  |  |
|  | Missing | 19 (1.8) | 11(0.9) | 0 | 0.1 | 0 | 0.1 | 0 | 0.1 | 19 (1.8) | 11(0.9) |  |  |  |  |  |
| Education attainment | Lower^a^ | 115 (10.6) | 128(10.6) | 86(10.6) | 93(10) | 81(11.5) | 85(9.5) | 54(11.1) | 51(8.1) | 115 (10.6) | 128(10.6) | 0.952 | 0.844 | **<0.05** | 0.230 | 0.952 |
|  | Higher^b^ | 742(68.4) | 819(67.9) | 594(73.2) | 679(73) | 536(76.4) | 660(73.7) | 372(76.7) | 494(78.8) | 742(68.4) | 819(67.9) |  |  |  |  |  |
|  | Missing | 228 (21) | 260 (21.5) | 131(16.2) | 158 (17) | 85(12.10 | 151 (16.9) | 59 (12.2) | 82 (13.1) | 228 (21) | 260 (21.5) |  |  |  |  |  |
| Employment | Not worked | 304(28) | 267(22.12) | 249(30.7) | 231(24.8) | 230 (32.8) | 219(24.4)) | 163(33.6) | 148(23.6) | 304(28) | 267(22.12) | **<0.001** | **<0.001** | **<0.001** | **<0.001** | **<0.001** |
|  | Worked | 647(59.6) | 817(67.7)) | 504(62.2) | 664(71.4) | 440(62.7) | 645(72) | 304(62.7) | 462(73.7) | 647(59.6) | 817(67.7)) |  |  |  |  |  |
|  | Missing | 134 (12.4) | 123 (10.2) | 58 (7.2) | 35 (3.8) | 32 (4.6) | 32 (3.6) | 18 (3.7) | 17(2.7) | 134 (12.4) | 123 (10.2) |  |  |  |  |  |
| Risk transmission | Vaginal | 744(68.6) | 774(64.1) | 565(69.7) | 610(65.6) | 509(72.5) | 582(65) | 352(72.6) | 423(67.5) | 744(68.6) | 774(64.1) | **<0.001** | **<0.001** | **<0.001** | **<0.001** | **<0.001** |
|  | Anal | 139(12.8) | 249(20.6) | 100(12.3) | 185(19.9) | 65(9.3) | 181(20.2) | 49(10.1 | 127(20.3) | 139(12.8) | 249(20.6) |  |  |  |  |  |
|  | Others^c^ | 89(8.2) | 92(7.6) | 77(9.5) | 68(7.3) | 60(8.6) | 66(7.4) | 39(8) | 48(7.7) | 89(8.2) | 92(7.6) |  |  |  |  |  |
|  | Missing |  |  | 69(8.5) | 67(7.2) | 68 (9.7) | 67 (7.5) | 45 (9.3) | 29 (4.6) |  |  |  |  |  |  |  |
| Test approach | PITC | 663(61.1) | 725(60.2) | --- | --- | --- |  | --- | --- | 663(61.1) | 725(60.2) | 0.389 |  |  |  | 0.389 |
|  | VCT | 285(26.3) | 306(25.4) | --- | --- |  |  | --- | --- | 285(26.3) | 306(25.4) |  |  |  |  |  |
|  | Missing | 137 (12.6) | 176(14.6) | --- | --- |  |  | --- | --- | 137 (12.6) | 176(14.6) |  |  |  |  |  |
| Baseline Clinical staging | I | --- | --- | 92(11.3) | 80(8.6) | 32(4.6) | 68(6.6) | 28(5.8) | 50(8) | 92(8.5) | 80(6.6) | --- | **<0.05** | **<0.05** | 0.433 | **<0.05** |
|  | II | --- | --- | 106 (13.1) | 126 (13.6) | 84(12) | 116(13) | 73 (15.1) | 96 (15.3) | 106 (9.8) | 126 (10.4) |  |  |  |  |  |
|  | III | --- | --- | 360 (44.4) | 457(49.1) | 360(51.3) | 457(51) | 254 (52.6) | 338(53.9) | 360 (33.2) | 457(37.9) |  |  |  |  |  |
|  | IV | --- | --- | 128 (15.8) | 165(17.7) | 128(18.2) | 165(18.4) | 86(17.7) | 94(15) | 128 (11.8) | 165(13.7) |  |  |  |  |  |
|  | Missing |  |  | 125 (15.4) | 102 (11) | 98 (14) | 90 (10) | 44 (9.1) | 49 (7.8) | 399 (36.8) | 379 (31.4) |  |  |  |  |  |
| Baseline CD4 cells/mm3 | Median | --- | --- | 120(32-265) | 115(31-276) | 112(31-269) | 98(29-223) | 97(30-210) | 120(35-275) | 120(32-265) | 115(31-276) |  |  | 0.073 | **<0.05** | 0.855 |
|  | < 350 |  |  | 493 (60.8) | 601(64.6) | 493(70.2) | 599 (66.9) | 385 (79.4) | 474 (75.6) | 493(45.4) | 601(49.8) |  | 0.234 | **<0.05** | 0.078 | 0.102 |
|  | >350 |  |  | 88(10.9) | 96 (10.3) | 41 (5.8) | 84(9.4) | 33 (6.8) | 67 (10.7) | 88(8.1) | 96(8) |  |  |  |  |  |
|  | Missing |  |  | 230 (28.4) | 233 (25.1) | 168 (23.9) | 213(23.8) | 67(13.8) | 86 (13.7) | 504(46.5) | 510 (43.3) |  |  |  |  |  |
| Treatment Indication | Baseline Clinical staging III or IV or CD4 cells count ≤ 350/mm3 | --- |  | --- | --- | --- | --- | 468(96.5) | 559(89.2) | 468(43.1) | 559(46.3) |  |  | **<0.001** | **<0.001** | **<0.001** |
|  | SUFA criteria and others |  |  |  |  |  |  | 16(3.3) | 60(9.6) | 16(<2) | 60(5) |  |  |  |  |  |
|  | Missing |  |  |  |  |  |  | 1(<1) | 2(<2) | 601(55.4) | 588(48.7) |  |  |  |  |  |
| ARV drug initiation | ZDV (300) + 3TC (150) + NVP (200 |  |  |  |  | --- |  | 259(53.4) | 113(18) | 259(23.9) | 113(9.4) |  |  |  | **<0.001** | **<0.001** |
|  | ZDV (300) + 3TC (150) + EFV (600) |  |  |  |  |  |  | 85(17.5) | 32(5.1) | 85(7.8) | 32(2.7) |  |  |  |  |  |
|  | TDF (300) + 3TC (150) + NVP (200) |  |  |  |  |  |  | 34(7) | 34(5.4) | 34(3.1) | 34(2.8) |  |  |  |  |  |
|  | TDF (300) +3TC (150) +EFV (600) |  |  |  |  |  |  | 90(18.6) | 101(16.1) | 90(8.3) | 101(8.4) |  |  |  |  |  |
|  | TDF (300) +3TC (300) +EFV (600) |  |  |  |  |  |  | 7(<2) | 334(53.3) | 7(<1) | 334(27.7) |  |  |  |  |  |
|  | Others |  |  |  |  |  |  | 3(<1) | 3(<1) | 3(<1) | 3(<1) |  |  |  |  |  |
|  | Missing |  |  |  |  |  |  | 7(<2) | 10(<2) | 607(55.9) | 590(48.9) |  |  |  |  |  |
| District | Medan | 552 (50.9) | 624(51.7) | 442(54.5) | 500 (53.8) | 369(52.6) | 479(53.5) | 265(54.6) | 347(55.3) | 552 (50.9) | 624(51.7) | 0.694 | 0.758 | 0.722 | 0.815 | 0.694 |
|  | Batam | 533 (49.1) | 583(48.3) | 369 (45.5) | 430 (46.2) | 333(47.4) | 417(46.5) | 220(45.4) | 280(44.7) | 533 (49.1) | 583(48.3) |  |  |  |  |  |
| Time | 2013 | 552(50.9) | 0 | 442 (54.5) | 0 | 369 (52.6) | 0 | 265(54.6) | 0 | 552(50.9) | 0 | **<0.001** | **<0.001** | **<0.001** | **<0.001** | **<0.001** |
|  | 2014 | 533 (49.1) | 624(51.7) | 369 (45.5) | 500 (53.8) | 333 (47.4) | 479 (53.5) | 220(45.4) | 347(55.3) | 533 (49.1) | 624(51.7) |  |  |  |  |  |
|  | 2015/16 | 0 | 583(48.3) | 0 | 430 (46.24) | 0 | 417 (46.5) | 0 | 280(44.7) | 0 | 583(48.3) |  |  |  |  |  |
|  |  |  |  |  |  |  |  |  |  |  |  |  |  |  |  |  |

^a^no school/primary school; ^b^ high school/higher education; ^c^PWID/ bisexual/perinatal/blood transfusion/occupational

*Pearson chi-square test or Mann-Whitney tes
