# Supplementary material for: Changes in the HIV continuum of care following expanded access to HIV testing and treatment in Indonesia: A retrospective population-based cohort study
Source: PLoS One. 2020 Sep 11;15(9):e0239041. doi: 10.1371/journal.pone.0239041 (PMC7485792; doi:10.1371/journal.pone.0239041)
Supplement: S4 Table — (DOCX) [file pone.0239041.s004.docx]

Supplementary table 4. Deaths before and after receiving treatment amongst newly diagnosed HIV cases pre and post SUFA intervention

|  | **Pre-SUFA n (%)**  (N=117) | **Post-SUFA n (%)**  (N=148) | **P-value^1^** |
| --- | --- | --- | --- |
| Before receiving treatment | 72 (61.5) | 100 (67.6) |  |
| After receiving treatment | 45 (38.5) | 48 (32.4) | 0.307 |

^1^From chi-squared test of association
