# Supplementary material for: Changes in the HIV continuum of care following expanded access to HIV testing and treatment in Indonesia: A retrospective population-based cohort study
Source: PLoS One. 2020 Sep 11;15(9):e0239041. doi: 10.1371/journal.pone.0239041 (PMC7485792; doi:10.1371/journal.pone.0239041)
Supplement: S5 Table — (DOCX) [file pone.0239041.s005.docx]

**Supplementary table** **5. Eligibility criteria used amongst eligible persons.**

| Eligibility criteria | Pre SUFA (N=702)  N (%) | Post-SUFA (N=896)  N (%) | **P-value^1^** |
| --- | --- | --- | --- |
| ≤ 350 CD4 or Clinical staging 3 or 4 | 673 (95.9) | 811 (90.5) |  |
| CD4 >350 or stage 1 or 2 or KAP | 29 (4.13) | 85 (9.5) | <0.001 |

^1^From chi-squared test of association
