# Supplementary material for: Changes in the HIV continuum of care following expanded access to HIV testing and treatment in Indonesia: A retrospective population-based cohort study
Source: PLoS One. 2020 Sep 11;15(9):e0239041. doi: 10.1371/journal.pone.0239041 (PMC7485792; doi:10.1371/journal.pone.0239041)
Supplement: S6 Table — (DOCX) [file pone.0239041.s006.docx]

Supplementary table 6. Treatment indication amongst patients initiated for treatment

| Treatment indication | **Pre-SUFA n (%)**  N=485 | **Post-SUFA n (%)**  N=627 | **P-value^1^** |
| --- | --- | --- | --- |
| Low CD4 count or advanced clinical condition | 468 (96.5) | 559 (89.2) |  |
| Within the SUFA population | 16 (3.3) | 60 (9.6) |  |
| Missing data | 1 (<0.1) | 8 (<0.2) | <0.001 |

^1^From Fisher’s Exact test
